# Evaluation of the content variation of anthraquinone glycosides in rhubarb by UPLC-PDA

**DOI:** 10.1186/1752-153X-7-170

**Published:** 2013-10-26

**Authors:** Zhe Wang, Pei Ma, Lijia Xu, Chunnian He, Yong Peng, Peigen Xiao

**Affiliations:** 1 Institute of Medicinal Plant Development, Chinese Academy of Medical Sciences, Peking Union Medical College, 151 Malianwa North Road, Beijing 100193, People’s Republic China; 2 Key Laboratory of Bioactive Substances and Resources Utilization of Chinese Herbal Medicine, Ministry of Education, 151 Malianwa North Road, Beijing 100193, People’s Republic China; 3 School of Pharmaceutical Sciences, Changchun University of Chinese medicine, Changchun People’s Republic China

**Keywords:** Rhubarb, UPLC-PDA, Anthraquinone glycoside, Quantitative analysis, Environmental factor

## Abstract

**Background:**

Rhubarb is an important Chinese medicinal herb with a long history of over 2000 years and has been commonly used as a laxative. It is the radix and rhizome of *Rheum officinale* Baill., *R. palmatum* L. and *R*. *tanguticum* Maxim, all of which are mainly distributed in a broad region in the Tibetan plateau. Anthraquinone glycosides are a series of major active ingredients found in all three species. They are key intermediates in the anthraquinone secondary metabolism and the sennnoside biosynthesis. The variation of the anthraquinone glycoside content in rhubarb in response to specific factors remains an attractive topic.

**Results:**

A simple and sensitive Ultra Performance Liquid Chromatography with Photo-Diode Array (UPLC-PDA) detector was developed for the simultaneous determination of six anthraquinone glycosides in rhubarb, *i.e.*, aloeemodin-8-*O*-glucoside, rhein-8-*O*-glucoside, chrysophanol-1-*O*-glucoside, emodin-1-*O*-glucoside, chrysophanol-8-*O*-glucoside, emodin-8-*O*-glucoside. Twenty-seven batches from three species were submitted to the multi-component analysis. The results showed that the anthraquinone glycoside content varied significantly even within the same species. The results showed that the anthraquinone glycoside content varied significantly within the same species but not between different species. The PCA and content analysis results confirmed that the plant species has no obvious effect on the content variation. Neither was any significant correlation observed between the anthraquinone glycoside content and the geographic distribution of the rhubarb. Through correlational analysis, altitude was found to be the main factor that affects the anthraquinone glycoside content in rhubarb. Rhubarb grown at higher altitude has higher anthraquinone glycoside content.

**Conclusions:**

This work provides a rapid, sensitive and accurate UPLC-PDA method for the simultaneous determination of six anthraquinone glycosides in rhubarb. The anthraquinone glycoside content varied significantly within the same species. The relationship of the anthraquinone glycoside content with plant species, geographic distribution and altitude were studied using correlational analysis, principal component analysis and spatial autocorrelation analysis through SPSS and ArcGIS. Plant species and geographic distribution were found not to affect the content of the six anthraquinone glycosides in rhubarb. The variations in the anthraquinone glycoside content were primarily due to the different altitude where the plant was grown.

## Background

Rhubarb has been clinically used for at least 2000 years as an antibacterial or laxative agent. In the Chinese Pharmacopoeia, rhubarb is list as the root of *Rheum officinale* Baill., *R. palmatum* L. and *R. tanguticum* Maxim [1]. These three species are mainly grown in the mountainous and desert regions of about 1200–4000 m a.s.l. in northwestern China and adjacent areas [2]. The extensive phytochemical research on rhubarb has led to the isolation and identification of about 200 chemical compounds, among which anthraquinone and its derivatives have been considered as the main active ingredients. For example, emodin and rhein are typical free anthraquinones that have strong antibacterial activity and sennoside A is a bianthrone derivative that has purgative activity [3]. The activity of rhubarb is often evaluated based on its anthraquinone content. The Chinese Pharmacopoeia specifies that the total anthraquinone content in rhubarb should not be less than 1.5%, and the Japanese Pharmacopoeia requires that the content of sennoside A must be higher than 0.25% [4]. Our previous study [5] found that although anthraquinones existed in all rhubarb samples, some rhubarb samples had only trace amount of sennoside A and sennoside B and are thus inactive. This prompted us to examine the anthraquinone secondary metabolites and the sennoside biosynthesis. The same molecular skeleton of anthraquinone and bianthrone indicates the existence of a secondary metabolism process through which anthraquinone gradually transforms into bianthrone, where anthraquinone glycoside serves as a key intermediate.

It is known that genetic and environmental factors and their interactions affect the pharmaceutically important secondary metabolites in medicinal plants [6]. A variety of environmental factors, such as season, altitude, radiation, and soil nutrition, have been proven to significantly influence the secondary metabolite profile [7-11].

A number of studies have evaluated the content of active ingredients, in rhubarb [12,13] and other medicinal plants[14] using HPLC. For the literature review, we found most of these researches focus on developing or optimization the analytical methods to determination the free anthraquinones. But until now, a systematic study on determination the anthraquinone glycoside in rhubarb was unavailable.

Based on the content of anthraquinones in rhubarb, pharmacologist carried out the research on the effect of different factors and the accumulation of ingredients. Temperature was found to significantly influence the contents of chrysophanol and physicon in rhubarb, whereas sunlight and precipitation had no significant influence [15]. Plant species research indicated that the anthraquinone content varied among different species and the sample of *R. officinale* from Sichuan had the highest anthraquinone content [16]. Plant samples cultivated for 5 years also had much higher anthraquinones content than those cultivated for 3 years [17]. Nevertheless, these works largely focused on environmental factors, ignoring the fact that anthraquinones are the primary metabolite and their content variation may depend on genetic factors that determine different metabolic pathways.

Six anthraquinone glycosides were previously isolated from rhubarb and some exhibited potential antioxidative activity and significant protective effects on cerebral ischemic injury [18]. Quantitative analysis of these ingredients is needed for dietary and pharmaceutical purposes. To date, the researches that determine the anthraquinone glycoside content in rhubarb mainly employ a multi-component analytic method to ensure clinical medication safety [19-21]. The content variation of anthraquinone glycoside in rhubarb reflects how the living environment affects the anthraquinone secondary metabolism and the accumulation of the active ingredient sennoside. However, to date, the content variation of anthraquinone glycosides in rhubarb and their influence factors have not been well studied.

This paper uses UPLC-PDA to determine the content of six anthraquinone glycosides in rhubarb. This method was applied to determine 27 samples from 3 species. The assay results were analyzed to evaluate the impact of plant species, geographic distribution and altitude on the anthraquinone glycoside content in rhubarb.

### Experimental

#### Plant materials

27 batches of wild rhubarb from 3 Rheum species were collected in China from the Gansu province, Qinghai province, Sichuan province and Yunnan province and Tibet, respectively.

Table 1 lists the details of each sample, including location, longitude, latitude and altitude. All samples were authenticated by Professor Min Li, Chengdu University of Traditional Chinese Medicine. The voucher specimens were deposited at the Herbarium of Institute of Medicinal Plant Development, Chinese Academy of Medical Sciences & Peking Union Medical College, Beijing, China.

**Table 1:** Detailed Information of all sampling sites and the content of six anthraquinone glycosides in rhubarb (mg/g) (n=3)

**Sample**	**Location**	**Longitude**	**Latitude**	**Altitude (m)**	**AE8G**	**R8G**	**E1G**	**C1G**	**C8G**	**E8G**	**Total**	**Mean ± SD**
*R. tanguticum*												
DH01	Maoxian, Sichuan	103.5449	32.16389	2808	10.63	tr	1.99	7.32	13.42	5.19	38.55	46.21 ± 19.25
DH02	Datong, Qinghai	103.6258	32.77226	2771	2.30	7.98	1.29	3.39	3.15	0.50	18.61	
DH03	Songpan, Sichuan	101.561	37.15853	3080	10.98	16.63	2.23	5.73	13.91	10.00	59.48	
DH04	Aba, Sichuan	102.2242	33.61313	2621	11.97	7.40	3.08	8.48	8.73	3.29	42.95	
DH05	Maqu, Gansu	101.8336	36.62261	3440	12.22	9.84	3.82	9.15	11.37	11.11	57.51	
DH06	Aba, Sichuan	101.8336	34.62262	2235	5.82	12.44	1.71	4.19	7.17	4.69	36.02	
DH07	Xining,Qinghai	101.6145	34.73594	2492	5.83	9.26	3.46	5.95	3.18	2.28	29.96	
DH08	Henan, Qinghai	103.1498	36.96756	3534	14.73	7.36	2.38	8.48	13.55	1.92	48.42	
DH09	Xunhuan, Qinghai	99.71639	27.8248	3659	11.94	6.26	1.82	15.46	46.99	1.92	84.39	
*R. officinale*												
DH10	Mianyang, Sichuan	104.505	31.72728	1295	tr	2.39	tr	tr	tr	tr	2.39	26.30 ± 22.98
DH11	Yulong, Yunan	99.46139	27.19084	2553	3.92	5.20	1.14	3.82	1.87	1.65	17.60	
DH12	Songpan, Sichuan	102.0491	33.9737	2621	5.08	7.06	0.98	3.75	3.68	0.42	20.97	
DH13	Chengdu, Sichuan	103.5372	30.98669	1574	4.71	7.48	1.73	4.76	4.05	3.61	26.34	
DH14	Kangding, Sichuan	97.17954	29.95119	3110	9.00	16.02	4.73	15.97	7.40	11.07	64.19	
*R. palmatum*												
DH15	Dangchang, Gansu	104.2187	34.23639	2243	1.84	6.07	0.66	1.72	5.08	1.33	16.70	35.54 ± 22.22
DH16	Dangchang, Gansu	104.9304	33.96551	2476	2.23	2.73	0.45	3.13	5.27	1.07	14.88	
DH17	Tianzhu, Gansu	102.9986	37.31148	2413	3.13	4.65	1.20	5.21	2.15	6.73	23.07	
DH18	Zeku, Qinghai	102.7261	35.61356	2946	2.21	6.17	tr	2.75	2.99	0.95	15.07	
DH19	Shangri-la , Yunan	102.7132	35.64171	3288	13.67	9.85	3.69	6.23	6.95	7.32	47.71	
DH20	Changdu, Tibet	97.17953	31.14529	3388	10.42	11.60	5.24	6.26	2.20	7.20	42.92	
DH21	Kangding, Sichuan	101.9349	29.95033	3854	18.33	10.27	5.46	14.79	8.81	18.33	75.99	
DH22	Minxian, Gansu	104.037	34.43876	2318	16.01	15.59	4.10	7.51	15.95	9.81	68.97	
DH23	Dangchang, Gansu	104.6847	33.95438	1728	2.33	1.87	tr	2.66	3.98	0.86	11.70	
DH24	Huzhu, Qinghai	102.9587	36.84461	2534	6.42	6.67	1.41	6.05	9.64	3.08	33.27	
DH25	Kangding, Sichuan	103.5449	32.16389	3110	8.72	10.62	4.63	17.03	8.10	7.90	57.00	
DH26	Tianzhu, Gansu	103.8949	31.6955	2593	7.15	13.70	2.59	4.07	6.14	8.72	42.37	
DH27	Lixian, Gansu	103.6258	32.77226	1895	1.74	2.81	tr	2.91	4.97	tr	12.43	

tr: below the LOQ.

#### Apparatus and reagents

**Figure 1:**
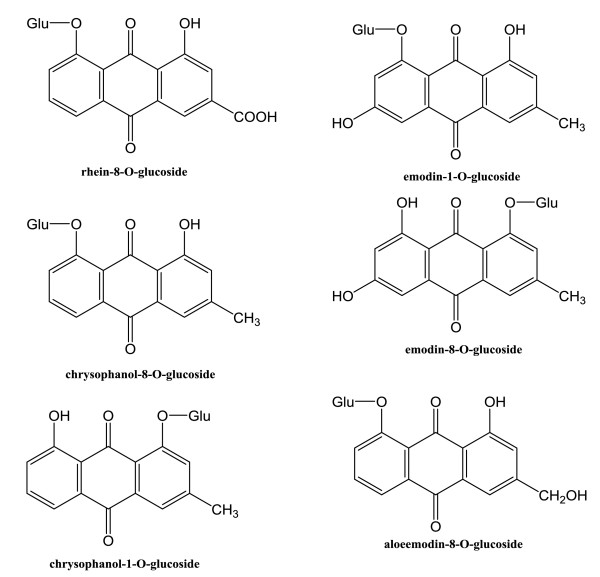
Molecular structure of the six anthraquinone glycosides.

**Figure 2:**
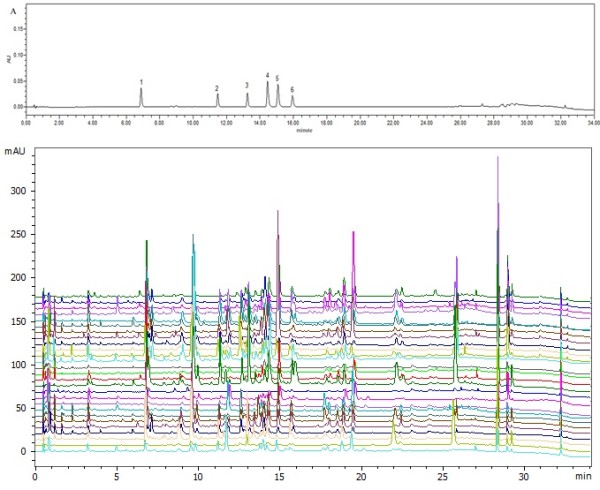
**UPLC-PDA chromatograms of the six anthraquinones and the samples.** Peaks labeled from 1 to 6 are AE8G, R8G, E1G, C1G, C8G, E8G.

Chromatographic analysis was performed on an Acquity UPLC system (Waters Corporation, Milford, USA) equipped with an online vacuum degasser, a quaternary pump, an autosampler, a temperature adjustable column compartment, and a Photo-Diode Array (PDA) detector, which was connected to Empower software. Chromatographic separation was performed on an Acquity UPLC BEH C18 column (2.1 × 100 mm, 1.7 μm; Waters Corporation). The column temperature was set at 35°C and the sample temperature was maintained at 4°C. HPLC-grade methanol and acetonitrile was purchased from Honeywell B&J Brand (Sleeze, Germany). Acetic acid (HPLC grade) was obtained from CNW Technologies GmbH (Dusseldorf, Germany). High purity water was obtained from Wahaha Co., Ltd. (Hangzhou, China). Analytical grade solvents for extraction and chromatography were purchased from Beijing Beihua Fine Chemicals Co. Ltd. (Beijing, China). The six standard compounds, aloeemodin-8-*O*-glucoside (AE8G), rhein-8-*O*-glucoside (R8G), emodin-1-*O*-glucoside (E1G), chrysophanol-1-*O*-glucoside (C1G), chrysophanol-8-*O*-glucoside (C8G), emodin-8-*O*-glucoside (E8G), were purchased from Chengdu Mansite Pharmaceutical Co. (Chengdu, China). Figure 1 shows the molecular structure of the six anthraquinone glycosides. All reference compounds had >98% purity (Figure 2) as confirmed by HPLC analysis.

### Quantitative analysis of anthraquinone glycoside by UPLC-PDA

#### Sample preparation

Approximately 0.05 g of finely ground dried rhubarb (filtered through a 40-mesh sieve) was extracted with 10 mL 80% methanol by ultrasonication at room temperature for 30 min. After centrifugation, the extracted solution was passed through a 0.22 μm syringe filter and an aliquot (1 μL) of the filtrate was injected into UPLC for analysis.

#### Standard preparation

All six reference standards were dissolved by 80% methanol to final concentrations of 0.105 mg/mL of AE8G, 0.208 mg/mL of R8G, 0.106 mg/mL of E1G, 0.209 mg/mL of C1G, 0.212 mg/mL of C8G, 0.124 mg/mL of E8G.

#### HPLC conditions

The components were quantified based on the peak areas at 280 nm in the UV spectrum. Elution was carried out at a flow rate of 0.5 mL/min. The mobile phase consisted of 0.5% (v/v) acetic acid in water (A) and acetonitrile (B) with a gradient program of 4%–10% (B) at 0–2 min, 10%–15% (B) at 2–6.5 min, 15% (B) at 6.5–8 min, 15%–20% (B) at 8–12 min, 20%–26.5% (B) at 12–20 min, 26.5%–30% (B) at 20–23 min, 30%–35% (B) at 23–25 min, 35%–55% (B) at 25–27 min, 55%–70% (B) at 27–30 min, 70%–98% (B) at 30–32 min.

## Results and discussion

### Optimization of quantitative analysis of anthraquinone glycoside

The extraction procedure was optimized to obtain high anthraquinone glycoside yield. Methanol, 80% methanol, 60% methanol and 40% methanol were used as extraction solvents (Additional file 1: Table S1), among which 80% methanol showed the best extraction efficiency for the six anthraquinone glycosides and was thus selected as the extraction solvent. Ultra-sonic extraction was used due to it fits to extract of multiple chemical compounds in medicinal plants [22,23]. The optimal extraction time was determined by comparing the peak areas of the marker compounds obtained after extraction for 15, 30, 45 and 60 min (Additional file 1: Table S2). The marker compounds showed no further increase after extraction for 30 min. Therefore, the optimized extraction conditions were ultra-sonic extraction by 80% methanol for 30 min.

### HPLC method validation

Linear regression analysis was performed by the external standard method using a series of standard solutions of the individual compounds in methanol at appropriate concentrations. All calibration curves demonstrated good linearity (*r*^2^ ≥ 0.9994). The linear ranges, calibration equation, determination coefficients (r^2^), LOD and LOQ for the analyses of the six compounds are summarized in Table 2. All RSDs of the areas of the characteristic peak in the precision and reproducibility tests were below 1.82% and 3.17%, respectively. Meanwhile, the RSD of the peak areas in the sample stability test was less than 2.58%. The recovery of compounds ranged in 96.53%–102.37% (RSD less than 3.57%), indicating that the method has a high accuracy. The above assay results indicate that the new UPLC-PDA method is accurate, reproducible, precise and sensitive enough for the simultaneous quantitative evaluation of anthraquinone glycosides in rhubarb. Until now, there are few systems suitable for the multi-component analysis of anthraquinone glycosides in rhubarb. Our method fills the gap by providing a convenient means in the future research of rhubarb.

**Table 2:** Calibration curves, LOD and LOQ data of six anthraquinone glycosides by UPLC-PDA

**Analytes**	**Calibration curves**	**r**^**2**^	**Linear range (μg/mL)**	**LOD (μg/mL)**	**LOQ (μg/mL)**
Aloeemodin-8-*O*-glucoside	y = 6082.4x − 3123.6	0.9998	5.00–420.00	0.08	0.25
Rhein-8-*O*-glucoside	y = 1959.5x + 1833.5	0.9999	9.89–830.40	0.06	0.17
Emodin-1-*O*-glucoside	y = 4298.9x + 7032.7	0.9998	5.07–425.60	0.1	0.29
Chrysophanol-1-*O*-glucoside	y = 4680.6x + 4860	0.9996	9.94–835.20	0.08	0.23
Chrysophanol-8-*O*-glucoside	y = 4359.2x + 5166.2	0.9997	10.08–846.40	0.05	0.14
Emodin-8-*O*-glucoside	y = 3328.6x + 15721	0.9994	5.90–496.00	0.22	0.67

### Comparison of anthraquinone glycoside content in rhubarb

The new UPLC-PDA method was applied to simultaneously determine six anthraquinone glycosides in 27 samples of three Rheum species collected from different areas. Representative chromatograms are shown in Figure 2 and the content of individual anthraquinone glycosides in the different samples are listed in Table 1.

The assay results showed that the six anthraquinone glycosides occurred commonly in most samples, although five samples (DH-1, DH-10, DH-18, DH-23, DH-27) lacked one or more anthraquinone glycosides. In all 27 samples, the content of all anthraquinone glycosides varied greatly, *i.e.*, 1.74–18.33 mg/g for AE8G, 1.87–35.55 mg/g for R8G, 0.45–18.61 mg/g for E1G, 1.72–17.03 mg/g for C1G, 1.87–46.99 mg/g for C8G, 0.42–18.33 mg/g for E8G. The assay results also showed that the samples from different species had obviously different levels of total anthraquinone glycosides. The samples from *R. tanguticum* had the highest total anthraquinone glycosides (mean ± SD, 46.21 ± 19.25 mg/g), followed by the samples from *R. palmatum* L. (mean ± SD, 35.54 ± 22.22 mg/g), and the samples from *Rheum officinale* had the lowest total anthraquinone glycosides (mean ± SD, 26.30 ± 22.98 mg/g). Sample DH-9 had the highest total anthraquinone glycosides (84.39 mg/g), which is approximately 35 times higher than DH-10 that had the lowest total anthraquinone glycosides (2.39 mg/g). The results indicated significant variations in the biosynthesis and accumulation of the anthraquinone glycosides.

### Plant species factor

The anthraquinone glycoside contents of the 27 samples determined by UPLC-PDA analysis were imported into Statistical Package for the Social Sciences (SPSS) 19.0. Correlational analysis and principal component analysis (PCA) were conducted (Additional file 2: Table S3). The results were visualized using the Spotfire 5.0 software.

**Figure 3:**
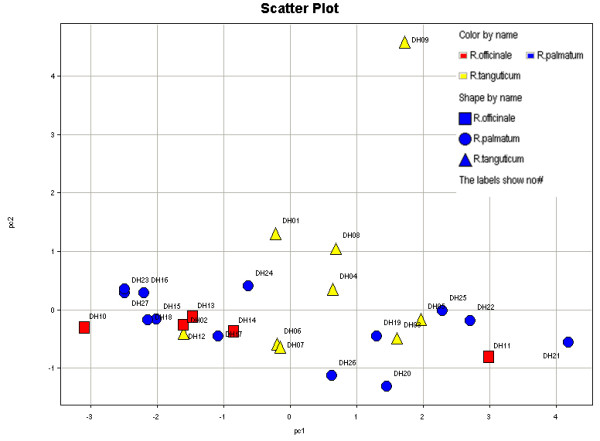
Score plot of principal component analysis (PC1-PC2) of 27 samples.

The potential usefulness of employing PCA with a combination of the key parameters and anthraquinone glycoside contents to classify the samples was investigated. Figure 3 shows a clear pattern for the three species. The first principal component (PC1) was the concentrations of compounds AE8G, R8G, E1G, C1G and E8G (scores 0.893, 0.726, 0.917, 0.834 and 0.858). The second principal component (PC2) was the concentration of compound C8G (scores 0.894) (Additional file 2: Table S4).

Although 27 samples from three species were tested, the PCA results failed to reflect the true plant species. All samples showed a ruleless distribution, indicating that the contents of the six anthraquinone glycosides are inadequate for distinguishing the different rhubarb species.

Nevertheless, some meaningful results were obtained where some patterns emerged. Among the nine samples (DH-01 to 09) from *R. tanguticum,* one sample (DH-09) was different from the others. A closer examination of the concentrations of the six anthraquinone glycosides suggested that the C8G content in DH-09 was the highest compared with other samples. Among the other eight samples, DH-06 and DH-07 were clustered into one group, and DH-03 and DH-05 were clustered into another group. These four samples were collected from completely different areas, but their anthraquinone glycoside contents were very similar. In addition, these four samples were collected from similar altitudes. Thus, we speculated that the altitude where the plant was grown may have a significant impact on the accumulation of anthraquinone glycosides.

Among the five samples (DH-10 to 14) from *R. officinale*, three samples (DH-12, 13 and 14) from different locations in the same province could be closely clustered into one group. DH-10 showed the lowest anthraquinone glycoside content among all 27 samples, and the content of C8G in DH-11 was less than in DH-12, DH-13 and DH-14.

The tested thirteen samples from *R. palmatum* could be evidently divided into two parts. The first part consisted of seven samples (DH-15, 16, 17, 18, 23, 24 and 27) that concentrated on the left side of PC1 of 0 and were close to each other. The second part consisted of the other six samples (DH-19, 20, 21, 22, 25 and 26) that concentrated on the right side of PC1 of 0 and had scattered distribution. The samples in the second part had much higher anthraquinone glycoside content than the samples in the first part. Four samples (DH-15, 16, 18 and 23) were clustered together closely, all of which had low anthraquinone glycoside content and were collected from an adjacent square in Gansu province. Thus, we speculated that the geographic distribution of the plant was also an environmental factor that may influence the accumulation of anthraquinone glycosides.

According to the results above, genetic factor does not appear to have significant correlation with the anthraquinone glycoside content in rhubarb, and plant species was eliminated to be the main influence factor of the variation in anthraquinone glycoside content. This is why according to the Chinese Pharmacopoeia, rhubarb from three Rheum species is used in clinical applications. Hence, the influence of environmental factors such as geographic distribution or altitude on the anthraquinone glycoside accumulation in rhubarb was explored next.

### Geographic distribution factor

Using SPSS 19.0, the correlation of the contents of anthraquinone glycosides in 27 samples and the latitude/longitude of the samples’ locations was examined. The spatial autocorrelation of the content of the six anthraquinone glycosides was done using ArcGIS 10.0.

The correlational analysis showed only weak correlations between latitude and the contents of AE8G and R8G (correlation factor −0.422 and −0.382, P < 95%) (Additional file 3: Table S5). In all other cases no correlation was found.

**Figure 4:**
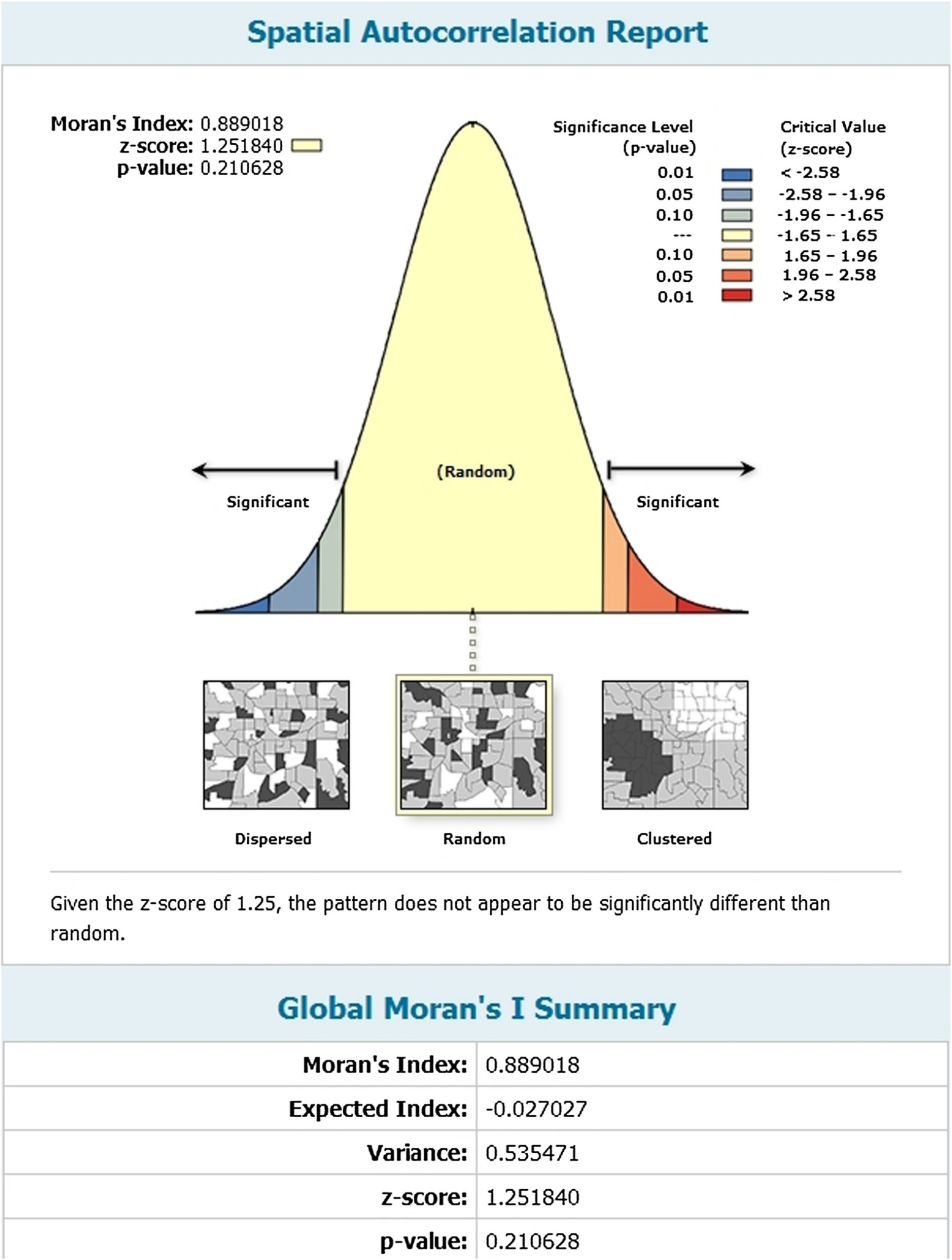
Spatial autocorrelation analysis results.

Spatial autocorrelation is a general statistical property of environmental variables that characterizes the correlation between signals at spatially nearby locations [24-26]. There are several indicators and methodologies to test the spatial autocorrelation. Moran’s I index is the most commonly used indicator and Z Test is used to validate the calculation of Moran’s index. The Z-score is a measure of standard deviation that verifies the statistical significance of the spatial autocorrelation analysis. The P-value is the significance level of the probability of completely random distribution. When the Z-score or P-value is statistically significant, the Moran's index is positive and the spatial distribution shows a pattern of aggregation; otherwise, the Moran's index is negative and the spatial distribution shows a dispersed pattern [27,28]. Figure 4 shows the spatial autocorrelation of total antharquinone glycosides. It can be seen that the P-value was 0.21, the Z-score was 1.25 and the Moran’s index was 0.90. Hence, the contents of total anthraquinone glycosides were entirely random and not affected by other spatial samples. It can be concluded that the content variations of the six anthraquinone glycosides in rhubarb were not affected by the geographic distribution of the plant.

### Altitude factor

The correlation between the anthraquinone glycoside contents in rhubarb samples and the altitude where their source plant was grown (determined by GPS) was analyzed using SPSS. Four anthraquinone glycosides, *i.e.*, AE8G, E1G, C1G, E8G, demonstrated remarkable correlation with altitude, with the correlation coefficients being 0.718, 0.631, 0.685, 0.533, respectively (P < 99%). Two other anthraquinone glycosides R8G and C8G showed weak correlation with correlation coefficients of 0.388 and 0.461 (P < 95%). The results suggested that altitude affects the biosynthesis and accumulation of anthraquinone glycosides in rhubarb.

**Figure 5:**
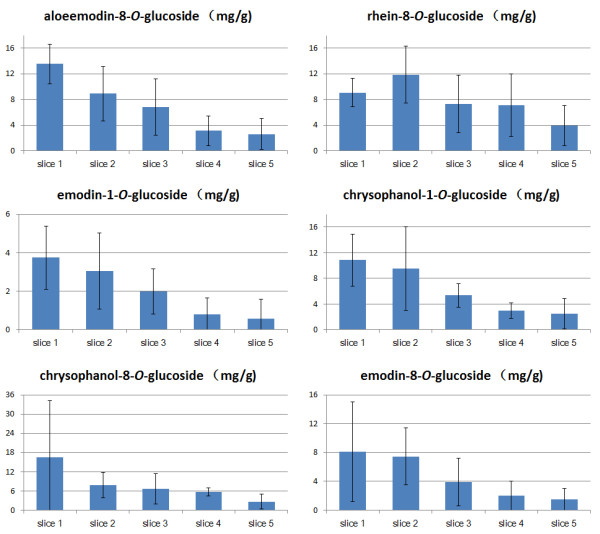
The individual anthraquinone glycoside content in each vertical slice.

For further investigation, vertical slices were used in ArcGIS and SPSS to show the characteristics of the anthraquinone glycoside contents of samples from similar altitude [24,29]. All samples were obtained from the altitude range of 1200–3900 m. After simulation and optimization, the total span was divided into five vertical slices, each covering 540 m height. This setting ensures that all vertical slices had at least 3 samples and the outcome was precise enough. Table 3 and Figure 5 show the individual and total anthraquinone glycoside content in each vertical slice.

**Table 3:** Samples in each vertical slice and the content of six anthraquinone glycosides in rhubarb (mg/g)

	**Altitude range (m)**	**Sample number**	**AE8G**	**R8G**	**E1G**	**C1G**	**C8G**	**E8G**	**Total**
**Slice 1**	3900–3360	DH-05,08,09,20,21	13.53±3.10	9.07±2.19	3.74±1.64	10.83±4.07	16.58±17.52	8.10±6.91	61.85±17.79
**Slice 2**	3360–2820	DH-03,11,18,19,25	8.92±4.24	11.86±4.42	3.06±1.98	9.54±6.50	7.87±3.92	7.45±3.94	48.69±19.73
**Slice 3**	2820–2280	DH-01,02,03,07,12,13,16,17,22,24,26,	6.79±4.40	7.29±4.47	1.97±1.17	5.33±1.86	6.65±4.73	3.89±3.29	31.93±15.89
**Slice 4**	2280–1740	DH-06,15,27	3.13±2.33	7.11±4.90	0.79±0.86	2.94±1.24	5.74±1.24	2.01±2.42	21.72±12.57
**Slice 5**	1740–1200	DH-10,14.23	3.36±2.93	3.91±3.10	0.58±1.00	2.47±2.39	2.68±2.32	1.49±1.89	14.49±11.98

Mono-factor analysis of variance was used to analyze the anthraquinone glycoside contents of samples in each vertical slice. According to the comparison results among the five slices, the content of AE8G, E1G and C1G of samples in each vertical slice showed significance (P < 95%). The F values for AE8G, E1G and C1G were 5.646, 3.781 and 4.671 (Additional file 4: Table S6). Table 3 shows the vertical slice results, including the height, the number of samples and the average content of each anthraquinone glycoside.

The vertical slice analysis revealed more information about the anthraquinone glycoside content at various altitudes. Firstly, the content variation was proved to be related to altitude. Samples at a high altitude always contained more anthraquinone glycosides. For example, the average content of anthraquinone glycosides in slice 1 was about 2 to 3 times higher than in slice 5. Secondly, the analysis revealed the best region for the accumulation of each anthraquinone glycoside. The best region to accumulate the anthraquinone glycosides was from 3360 to 3900 m, while for R8G the best altitude was 2820 to 3360 m. Lastly, the rhubarb samples collected at low altitude had remarkably decreased anthraquinone glycoside contents and consequently may be clinically inactive. The rhubarb samples obtained from below 2500 m had a total anthraquinone glycoside content of 24.25 ± 20.19 mg/g (mean ± SD), which was less than half of those obtained above 2500 m (45.12 ± 18.55 mg/g). The measurement results suggested that samples collected below 2500 m should be taken with double dose to ensure their efficacy.

## Discussion

As we previously mentioned, genetic and environmental factors and their interactions affect the pharmaceutically important secondary metabolites in medicinal plants. In our previous study [5], we revealed the variation of anthraquinones contents in the same bitch of samples were mainly caused by the genetic factor, and no relationship with environmental factors, *i.e.*, geographic distribution and altitude. In this study, the result shown that the anthraquinonese glycosides content were less variation in three species plants, it’s due to the these three rhubarb plants were derived from sect Rheum (Polygonacea), and with a close genetic information. While the anthraquinone glycoside content variations significantly within the same species revealed that the environmental factor played an important role in the anthraquinones glycoside production and accumulation. Our researches showed that the anthraquinone glycosides in rhubarb have a positive correlation with the altitude of their sampling location. Higher altitude could promote the accumulation of anthraquinone glycosides. Rhubarb is a typical alpine plant that can survive at altitude up to 4000 m a.s.l. The Tibetan plateau is under the influence of high altitude, latitude, mountains and glaciers, which results in unique regional climate features such as strong solar radiation, long sunshine time, low temperature and large intraday temperature spread. Commonly, temperature decreases with rising altitude, e.g., a drop of 0.6°C for an elevation of 100 meters. To avoid freezing injury caused by the rising altitude and ensure survival in the harsh environment, the plateau plants increase the content of soluble sugar, which is an important osmotic adjustment material [30-33]. These survey results showed that high altitude can induce the accumulation of soluble sugar in plants. Glucose is the most basic soluble sugar and the reactant in anthraquinone glycosylation. A high amount of glucose should boost the biosynthesis and accumulation of anthraquinone glycosides in rhubarb. Based on the abovementioned conditions, the content variation of anthraquinone glycosides was further speculated to relate with altitude increase, which lead to temperature drop and glucose accumulation, resulting in the difference of biosynthesis and accumulation of anthraquinone glycosides. This prediction was strongly confirmed by our research results.

## Conclusion

We here report a rapid, sensitive and accurate UPLC-PDA method for the simultaneous determination of six anthraquinone glycosides in rhubarb. The new method was successfully applied to simultaneously determine six anthraquinone glycosides in 27 rhubarb samples from three species. The content variation reflected some factors that affected the accumulation of anthraquinone glycosides during their secondary metabolism. Plant species, geographic distribution and altitude were examined as three main factors. Using correlational analysis, principal component analysis and spatial autocorrelation analysis through SPSS and ArcGIS, the plant species and geographic distribution were excluded as influencing factors. It was found that altitude may played an important role in the accumulation of anthraquinone glycosides in these 27 rhubarb samples, collected from the main distribution area of China. In the study, we get to the potential result that the contents of the six anthraquinone glycosides increased with rising altitude of their sampling location. We speculated that the best region for the accumulation of anthraquinone glycosides in rhubarb was at >3000 m a.s.l from the outcome. We also showed the large differences in the contents of the six anthraquinone glycosides among samples collected at different altitude.

## Abbreviations

UPLC: Ultra performance liquid chromatography; PDA: Photo-diode array; SPSS: Statistical product and service solutions; GIS: Geographic information system; PCA: Principal component analysis; AE8G: Aloeemodin-8-*O*-glucoside; R8G: Rhein-8-*O*-glucoside; E1G: Emodin-1-*O*-glucoside; C1G: Chrysophanol-1-*O*-glucoside; C8G: Chrysophanol-8-*O*-glucoside; E8G: Emodin-8-*O*-glucoside.

## Competing interests

The authors declare that they have no competing interests.

## Authors’ contributions

PX initiated and all authors designed the study. The samples were collected by ZW. The method developments were conducted by PM and LX. CH and YP contributed to the assay analysis. All authors have read and approved the final version of the manuscript.

## Supplementary Material

Additional file 1 HPLC results by different extraction methods. Click here for file

Additional file 2 PCA results of species analysis. Click here for file

Additional file 3 The correlation of anthraquinone glycoside and environment factors. Click here for file

Additional file 4 Mono factor analysis ofanthraquinone glycosides in each vertical slice. Click here for file
