# Supplementary material for: Evaluation of the content variation of anthraquinone glycosides in rhubarb by UPLC-PDA
Source: Chem Cent J. 2013 Oct 26;7:170. doi: 10.1186/1752-153X-7-170 (PMC3854541; doi:10.1186/1752-153X-7-170)
Supplement: Additional file 1 — HPLC results by different extraction methods. [file 1752-153X-7-170-S1.docx]

Table S1

HPLC results by different extraction solvents (mg/g)

| sample | Extraction solvents | AE8G | R8G | E1G | C1G | C8G | E8G | total |
| --- | --- | --- | --- | --- | --- | --- | --- | --- |
| DH-04 | methanol | 10.50 | 6.97 | 2.89 | 7.79 | 8.13 | 2.99 | 39.27 |
|  | 80% methanol | 11.90 | 7.35 | 3.04 | 8.52 | 8.67 | 3.29 | 42.77 |
|  | 60% methanol | 11.82 | 7.40 | 2.97 | 8.43 | 8.55 | 3.31 | 42.48 |
|  | 40% methanol | 11.35 | 7.17 | 2.92 | 8.14 | 8.42 | 3.28 | 41.28 |

Table s2

UPLC results by different extraction time (mg/g)

| sample | extraction times | AE8G | R8G | E1G | C1G | C8G | E8G | total |
| --- | --- | --- | --- | --- | --- | --- | --- | --- |
| DH-04 | 15 min | 11.77 | 7.29 | 2.92 | 8.31 | 8.43 | 3.22 | 41.94 |
|  | 30 min | 11.93 | 7.37 | 3.05 | 8.53 | 8.72 | 3.29 | 42.89 |
|  | 45 mim | 11.94 | 7.36 | 3.05 | 8.54 | 8.75 | 3.29 | 42.93 |
|  | 60 min | 11.94 | 7.37 | 3.07 | 8.56 | 8.74 | 3.28 | 42.96 |
