# Supplementary material for: Evaluation of the content variation of anthraquinone glycosides in rhubarb by UPLC-PDA
Source: Chem Cent J. 2013 Oct 26;7:170. doi: 10.1186/1752-153X-7-170 (PMC3854541; doi:10.1186/1752-153X-7-170)
Supplement: Additional file 2 — PCA results of species analysis. [file 1752-153X-7-170-S2.docx]

Table S3

Total variance explained of PCA

|  | | | | | | |
| --- | --- | --- | --- | --- | --- | --- |
| Component | Initial Eigenvalues | | | Extraction Sums of Squared Loadings | | |
|  | Total | % of variance | Cumulative % | Total | % of variance | Cumulative % |
| 1 | 3.790 | 63.173 | 63.173 | 3.790 | 63.173 | 63.173 |
| 2 | 1.165 | 19.409 | 82.583 | 1.165 | 19.409 | 82.583 |
| 3 | .465 | 7.749 | 90.331 | .465 | 7.749 | 90.331 |
| 4 | .310 | 5.159 | 95.490 | .310 | 5.159 | 95.490 |
| 5 | .214 | 3.564 | 99.054 | .214 | 3.564 | 99.054 |
| 6 | .057 | .946 | 100.000 | .057 | .946 | 100.000 |
| Extraction Method: Principal Component Analysis. | | | | | | |

Table S4

Component Matrixa ^a^

|  | | | | | | |
| --- | --- | --- | --- | --- | --- | --- |
|  | Component | | | | | |
|  | 1 | 2 | 3 | 4 | 5 | 6 |
| Zscore(AE8G) | .893 | .159 | -.065 | -.370 | -.154 | -.108 |
| Zscore(R8G) | .726 | -.401 | .541 | .126 | -.033 | -.045 |
| Zscore(E1G) | .917 | -.240 | -.162 | .055 | -.220 | .157 |
| Zscore(C1G) | .834 | .318 | -.236 | .371 | .017 | -.096 |
| Zscore(C8G) | .440 | .849 | .265 | -.039 | .081 | .090 |
| Zscore(E8G) | .858 | -.315 | -.123 | -.120 | .366 | .030 |
| Extraction Method: Principal Component Analysis. | | | | | | |
| a. 6 components extracted. | | | | | | |
