# Supplementary material for: Evaluation of the content variation of anthraquinone glycosides in rhubarb by UPLC-PDA
Source: Chem Cent J. 2013 Oct 26;7:170. doi: 10.1186/1752-153X-7-170 (PMC3854541; doi:10.1186/1752-153X-7-170)
Supplement: Additional file 3 — The correlation of anthraquinone glycoside and environment factors. [file 1752-153X-7-170-S3.docx]

Table S5

Correlation of anthraquinone glycoside and Latitude, Longitude and Altitude.

|  |  | | | | | | | | | | | |
| --- | --- | --- | --- | --- | --- | --- | --- | --- | --- | --- | --- | --- |
|  | | AE8G | R8G | E1G | C1G | C8G | E8G | Total | Latitude | Longitude | Altitude |  |
| AE8G | Pearson Correlation | 1 | .513^**^ | .788^**^ | .682^**^ | .503^**^ | .709^**^ | .886^**^ | .074 | -.422^*^ | .718^**^ |  |
|  | Significance （2-tailed） |  | .006 | .000 | .000 | .007 | .000 | .000 | .714 | .029 | .000 |  |
|  | N | 27 | 27 | 27 | 27 | 27 | 27 | 27 | 27 | 27 | 27 |  |
| R8G | Pearson Correlation | .513^**^ | 1 | .681^**^ | .400^*^ | .111 | .654^**^ | .636^**^ | .369 | -.382^*^ | .388^*^ |  |
|  | Significance （2-tailed） | .006 |  | .000 | .038 | .583 | .000 | .000 | .059 | .049 | .045 |  |
|  | N | 27 | 27 | 27 | 27 | 27 | 27 | 27 | 27 | 27 | 27 |  |
| E1G | Pearson Correlation | .788^**^ | .681^**^ | 1 | .728^**^ | .151 | .800^**^ | .774^**^ | .187 | -.304 | .631^**^ |  |
|  | Significance （2-tailed） | .000 | .000 |  | .000 | .453 | .000 | .000 | .349 | .123 | .000 |  |
|  | N | 27 | 27 | 27 | 27 | 27 | 27 | 27 | 27 | 27 | 27 |  |
| C1G | Pearson Correlation | .682^**^ | .400^*^ | .728^**^ | 1 | .552^**^ | .603^**^ | .849^**^ | .116 | -.082 | .685^**^ |  |
|  | Significance （2-tailed） | .000 | .038 | .000 |  | .003 | .001 | .000 | .566 | .683 | .000 |  |
|  | N | 27 | 27 | 27 | 27 | 27 | 27 | 27 | 27 | 27 | 27 |  |
| C8G | Pearson Correlation | .503^**^ | .111 | .151 | .552^**^ | 1 | .114 | .692^**^ | .221 | -.209 | .461^*^ |  |
|  | Significance （2-tailed） | .007 | .583 | .453 | .003 |  | .572 | .000 | .268 | .296 | .016 |  |
|  | N | 27 | 27 | 27 | 27 | 27 | 27 | 27 | 27 | 27 | 27 |  |
| E8G | Pearson Correlation | .709^**^ | .654^**^ | .800^**^ | .603^**^ | .114 | 1 | .736^**^ | .067 | -.367 | .533^**^ |  |
|  | Significance （2-tailed） | .000 | .000 | .000 | .001 | .572 |  | .000 | .741 | .059 | .004 |  |
|  | N | 27 | 27 | 27 | 27 | 27 | 27 | 27 | 27 | 27 | 27 |  |
| Latitude | Pearson Correlation | .074 | .369 | .187 | .116 | .221 | .067 | .233 | 1 | .292 | .260 |  |
|  | Significance （2-tailed） | .714 | .059 | .349 | .566 | .268 | .741 | .243 |  | .139 | .190 |  |
|  | N | 27 | 27 | 27 | 27 | 27 | 27 | 27 | 27 | 27 | 27 |  |
| Longitude | Pearson Correlation | -.422^*^ | -.382^*^ | -.304 | -.082 | -.209 | -.367 | -.375 | .292 | 1 | .112 |  |
|  | Significance （2-tailed） | .029 | .049 | .123 | .683 | .296 | .059 | .054 | .139 |  | .577 |  |
|  | N | 27 | 27 | 27 | 27 | 27 | 27 | 27 | 27 | 27 | 27 |  |
| Total | Pearson Correlation | .886^**^ | .636^**^ | .774^**^ | .849^**^ | .692^**^ | .736^**^ | 1 | .233 | -.375 | .731^**^ |  |
|  | Significance （2-tailed） | .000 | .000 | .000 | .000 | .000 | .000 |  | .243 | .054 | .000 |  |
|  | N | 27 | 27 | 27 | 27 | 27 | 27 | 27 | 27 | 27 | 27 |  |
| Altitude | Pearson Correlation | .718^**^ | .388^*^ | .631^**^ | .685^**^ | .461^*^ | .533^**^ | .731^**^ | .260 | .112 | 1 |  |
|  | Significance （2-tailed） | .000 | .045 | .000 | .000 | .016 | .004 | .000 | .190 | .577 |  |  |
|  | N | 27 | 27 | 27 | 27 | 27 | 27 | 27 | 27 | 27 | 27 |  |
|  | **. P<99%  *.P<95%. | | | | | | | | | | | |
