# Supplementary material for: Evaluation of the content variation of anthraquinone glycosides in rhubarb by UPLC-PDA
Source: Chem Cent J. 2013 Oct 26;7:170. doi: 10.1186/1752-153X-7-170 (PMC3854541; doi:10.1186/1752-153X-7-170)
Supplement: Additional file 4 — Mono factor analysis ofanthraquinone glycosides in each vertical slice. [file 1752-153X-7-170-S4.docx]

Table S6

Mono factor analysis of variance on the samples content of anthraquinone glycosides in each vertical slice

|  | AE8G* | R8G | E1G* | C1G* | C8G | E8G | Total* |
| --- | --- | --- | --- | --- | --- | --- | --- |
| F | 5.646 | 2.081 | 3.782 | 4.671 | 1.757 | 2.240 | 5.921 |
| P-value | 0.003 | 0.118 | 0.017 | 0.007 | 0.174 | 0.098 | 0.002 |
| F crit | 2.817 | 2.817 | 2.817 | 2.817 | 2.817 | 2.817 | 2.817 |

P<95%

* significance
